# Understanding the nature and impact of cognitive fluctuations and sleep disturbances in dementia with Lewy bodies: A qualitative caregiver study

**DOI:** 10.1177/20503121241271827

**Published:** 2024-10-09

**Authors:** Ellie Matterson, Gemma Wilson-Menzfeld, Kirsty Olsen, John-Paul Taylor, Greg J Elder

**Affiliations:** 1 Northumbria Sleep Research, Northumbria University, Newcastle upon Tyne, UK; 2 Faculty of Health and Life Sciences, Department of Nursing, Midwifery, and Health, Northumbria University, Newcastle upon Tyne, UK; 3 Translational and Clinical Research Institute, Newcastle University, Campus for Ageing and Vitality, Newcastle upon Tyne, UK

**Keywords:** Caregivers, cognitive fluctuations, dementia with Lewy bodies, sleep

## Abstract

**Objectives:**

Dementia with Lewy bodies is characterised by rapid fluctuations in attention, which are known as “cognitive fluctuations.” Despite the fact that cognitive fluctuations are considered to be a core dementia with Lewy bodies symptom, they are very difficult to define and measure using existing quantitative subjective measurement tools, which are typically completed by caregivers. Cognitive fluctuations are also likely to be influenced by various aspects of sleep, but this is as yet unexplored. The primary aim of this qualitative study was to investigate the phenomenology of cognitive fluctuations in dementia with Lewy bodies by understanding caregiver experiences.

**Methods:**

Seven caregivers of people with dementia with Lewy bodies completed one-to-one semistructured interviews, which were conducted by telephone. Caregivers were asked to describe the nature, frequency, duration and potential triggers of cognitive fluctuations that were experienced by the individual with dementia with Lewy bodies. Caregivers were also asked about the subjective sleep experience of the individual with dementia with Lewy bodies, and about their own sleep experiences. Interviews were audio recorded, transcribed verbatim and analysed using Thematic Analysis.

**Results:**

Caregivers reported that there was a great deal of individual variation in the frequency, duration and severity of cognitive fluctuations. Patient sleep disturbances, including excessive daytime sleepiness, nocturnal awakenings, restless legs and sleep apnoea, were common. However, the impact of sleep alterations or experiences upon the fluctuations was reported to be less clear. Caregivers also reported that their own sleep was negatively affected, potentially due to actively listening for overnight events and behaviours.

**Conclusions:**

Qualitatively, caregivers report that dementia with Lewy bodies cognitive fluctuations show large individual variations in terms of their frequency, duration and severity, but that subjectively, sleep may not consistently influence this symptom. Specific, caregiver-focussed interventions are likely to be necessary to maintain good sleep health in dementia with Lewy bodies caregivers.

## Introduction

Dementia with Lewy bodies (DLB) is the second most common type of dementia after Alzheimer’s dementia (AD) and accounts for between 15% and 20% of all dementia cases *post mortem*.^1,2^ DLB is a complex neurodegenerative condition, and individuals with DLB display a wide range of symptoms, including attentional and visuoperceptual deficits and visual hallucinations.^3^ One prominent and debilitating DLB symptom is that of cognitive fluctuations, which refer to marked variations in cognition and arousal. Cognitive fluctuations are characterised by interruptions in awareness, reduced alertness, transient episodes of confusion and by subsequent difficulties in communication.^4–7^ Up to 90% of individuals with DLB display cognitive fluctuations, and this symptom has a major impact upon activities of daily living, and their presence can exacerbate other distressing DLB symptoms such as visual hallucinations.^8,9^

Despite their high prevalence, and status as a core DLB symptom, cognitive fluctuations are extremely hard to define, and for this reason, their aetiology is currently poorly understood.^3,10^ It is very likely that sleep can affect this symptom, as the timing and duration of sleep, and occurrence of sleep deprivation, can influence subsequent cognitive performance.^11^ Additionally, common brain regions underpin both sleep and cognitive fluctuations.^12,13^ Moreover, the common DLB symptom of excessive daytime sleepiness may directly affect fluctuations due to DLB-specific circadian rhythm alterations, which modify the timing and quality of sleep.^14^ Whilst a range of subjective and objective measurement tools have been used to assess cognitive fluctuations, including caregiver questionnaires and the assessment of resting brain activity,^15,16^ there are questions regarding the suitability and accuracy of these techniques.^10^ Consequently, cognitive fluctuations remain difficult to measure both within a clinical and research context.

As the phenomenology of cognitive fluctuations is poorly understood, qualitative methodologies, where caregivers are interviewed, can be used to better understand this symptom. This is because qualitative methodologies can allow for the perspective and experience of the caregiver to be considered and explored.^1^

In comparison to quantitative methodologies, the use of caregiver interviews enables the collection of detailed, relevant information which cannot be collected using existing subjective questionnaire measures.^16^ Therefore, qualitative approaches can enable the phenomenology of cognitive fluctuations to be explored in more detail with caregivers, and qualitative information collected in this context can be used to develop improved clinical and research tools for DLB cognitive fluctuations. However, to date, no DLB studies have specifically investigated the phenomenology of cognitive fluctuations using a qualitative methodology. Additionally, this methodology allows for a more detailed examination of the impact of this symptom upon the caregiver experience, which is also difficult to qualitatively investigate using existing questionnaire measures. This is important because DLB places a significant level of burden upon caregivers, and the caregivers typically report greater levels of distress than the caregivers of people with AD or other types of dementia, even when there are similar levels of cognitive impairment.^17–19^ As the symptom of cognitive fluctuations specifically contributes to DLB caregiver burden,^7,18^ a more detailed understanding of the caregiver perspective can be used to inform the design of future interventions which are specifically designed to benefit caregivers. An interview approach can also allow for the exploration of whether caregivers consider aspects of patient sleep, such as poor nocturnal sleep quality or excessive daytime sleepiness, to specifically trigger or modify the frequency and/or severity of subsequent daytime fluctuations, given the common pathway underpinning sleep and cognitive fluctuations.

Therefore, the primary aim of this qualitative study was to explore the phenomenology of cognitive fluctuations in DLB. The secondary aims of the study were to examine if caregivers considered sleep, or sleep disturbances, to influence the nature of cognitive fluctuations, and to also understand the nature of habitual sleep patterns, sleep changes and sleep disturbances, and their impact upon people with DLB and their caregivers.

## Method

### Design

As this study aimed to focus on the lived experiences and observations of the caregivers of people with DLB in relation to their cognitive fluctuations and subjective sleep experiences, a qualitative design was appropriate for this study. This study was conducted as a pilot study alongside a larger ongoing quantitative study which had the specific aim of assessing cognitive fluctuations and sleep in DLB using multiple objective methods. For this reason, we drew upon pragmatism as the most appropriate methodology for the present qualitative study, as this supports the use of multiple philosophical positionings to assess a particular phenomenon.^20^ The study was approved by a United Kingdom National Health Service (NHS) Research Ethics Committee (East of England Research Ethics Committee; REC reference: 19/EE/0223). The study has been reported in line with the Consolidated Criteria for Reporting Qualitative Research (COREQ) guidelines.^21^

### Measures

Participants with DLB had their global cognitive function assessed using the Mini-Mental State Examination (MMSE).^22^ At the same time, caregiver participants provided a subjective estimate of the presence and severity of participant cognitive fluctuations using the Clinical Assessment of Fluctuation (CAF) scale,^23^ which assesses cognitive fluctuations over the previous month. For DLB participants, information was also obtained with regards to whether or not they routinely used cholinesterase inhibitors, or antidepressant, sleep or anti-Parkinsonian medication. These assessments were completed during participation in the larger quantitative study, which took place before caregiver interviews.

### Participants

A total of seven participants were interviewed. All participants were the caregivers of people who had a diagnosis of DLB in accordance with current diagnostic criteria^3^ and were recruited from the north-east of England. All DLB participants were verified as having a diagnosis by two experienced specialist Old Age Psychiatry clinicians who reviewed their medical records, in line with previous work.^24,25^ The inclusion criteria for participating in this study involved being the primary caregiver of an individual with DLB. There were no specific exclusion criteria.

The present sample size was not determined in advance, as caregiver participants were approached using convenience sampling, due to being previously known to the wider research team due to their ongoing participation alongside DLB participants in the larger quantitative study, and due to the pilot nature of the study. The individuals with DLB were recruited from specialist clinical services (e.g., memory clinics) in the north-east of England. Caregiver participants were initially approached by telephone or email by a familiar member of the research team, and no participants refused to take part in the study. All interviewees were given an information sheet and had the opportunity to ask questions before providing consent. Written informed consent was obtained from all participants before the study. Participants were interviewed between July 2021 and August 2022 and were not renumerated for their participation.

### Procedure

Caregivers of people with DLB were approached and consented for interviews. All interviews were semi-structured using an interview schedule (Supplemental Material 1), which was developed in relation to the main aims of the study. The interview schedule was not pilot-tested, but the design of the interview schedule was informed by our previous ongoing work with Lewy body dementia caregivers. Additionally, feedback from caregivers who were taking part in other DLB studies were used to help develop and improve the interview questions prior to their use. Caregiver participants were asked all interview questions.

During the interview, participants were asked to explain the nature and characteristics of the cognitive fluctuations that they observed, in response to interview questions. These characteristics specifically included the duration, features and the impact of cognitive fluctuations upon the individual with DLB, and the impact upon themselves as caregivers. Participants were also asked for their observations in relation to subjective sleep experienced by the individual with DLB.

All interviews were conducted by telephone. Interviews were conducted by a member of the research team (co-author EM; female), who holds an undergraduate degree in psychology and was a research assistant in Lewy body dementia at the time of participant interview. The interviewer did not have a prior relationship with study participants, and participants were made aware of the aims of the interview before providing informed consent. The researcher briefly discussed the symptom of cognitive fluctuations and ensured that caregivers understood what was meant by this term (i.e., rapid changes in attention and cognition demonstrated by the person with DLB) before beginning the interview.

All interviews (mean interview duration = 19.47 mins) were audio-recorded. Field notes were not made during the interview process, as all interviews were transcribed verbatim after the interview had ended. Transcripts were not returned to participants before further analysis.

### Data analysis

Interviews continued until data saturation were achieved. In this study, this referred to the point at which additional interviews did not provide any new information in relation to the study’s research question. Interview data were obtained from seven caregiver participants. Interview data were coded and analysed using inductive, reflexive, thematic analysis; thematic analysis was employed as the data analysis strategy due to its theoretical freedom.^26,27^ This study followed the six steps of thematic analysis (familiarity with the dataset, generating initial codes, developing themes, reviewing and adapting themes and producing the final report). Interviews were independently coded by EM and GE, and both themes and subthemes were derived from data. Themes and subthemes were considered to be patterns of shared meaning.^27^ All authors discussed and agreed the final themes and subthemes.

## Results

Caregiver and DLB participant demographic data are reported in Table 1. The majority of caregivers (70%) were females, and all caregivers were spouses of the individual with DLB. Most of the caregivers reported sharing a bedroom with DLB participants (57%). The participants with DLB displayed moderate levels of dementia (mean MMSE score = 23.43) and cognitive fluctuation severity (mean CAF score = 3). Most participants reported having physical co-morbidities (86%) and three (43%) participants reported having psychological co-morbidities (e.g., depression or anxiety).

**Table 1.** Caregiver and DLB participant information (*n* = 7).

Variable	Mean	SD
Caregiver		
Gender (male/female; *n* (%))	2M (28.6); 5F (71.4)	
Relationship to person with DLB		
Spouse	7 (100)	
Proportion of caregivers who share a bedroom with DLB participant (yes/no; *n*; %)	4Y (57.1); 3N (42.9)	
DLB participant		
Age (years)	72.43	6.13
Dementia duration (months)	48.67	28.02
MMSE	23.43	3.65
CAF	3.00	3.64
Participants with physical comorbidities (*n*; %)	6 (85.7)	
Participants with psychological comorbidities (*n*; %)	3 (43%)	
Participants taking cholinesterase inhibitors (*n*; %)	7 (100)	
Participants taking antidepressant medication (*n*; %)	2 (28.6)	
Participants taking Parkinsonian medication (*n*; %)	4 (57.1)	
Participants taking sleep medication (*n*; %)	1 (14.3)	

CAF: clinical assessment of fluctuations scale; DLB: dementia with Lewy bodies; MMSE: mini-mental state examination.

A total of two themes were generated from the qualitative interview data: (1) that the symptomology of cognitive fluctuations was inconsistent and (2) the presence of patient and caregiver subjective sleep disturbances. Each theme had multiple sub-themes.

## Theme 1: Inconsistent symptomology of cognitive fluctuations

The key finding with regards to this theme is that caregivers reported that there was a great deal of individual variation in the frequency and duration of the observed cognitive fluctuations, as well as in their apparent severity. As part of this theme, participants also discussed what they felt were possible ‘triggers’ for this symptom.

### Sub-Theme 1: Frequency, duration and severity of cognitive fluctuations

The key finding from this sub-theme was that caregivers reported that the frequency, duration and severity of cognitive fluctuations was inconsistent and appeared to change over time for many of the individuals with DLB. Specifically, individuals with DLB could go for days without appearing to experience this symptom, and for that reason, caregivers felt that they would not know what to expect on any given day. Caregivers also reported that within-day variations were apparent:

He could maybe have the odd day and he’ll be totally lucid all the time. Then he could have another day and he can be quite confused most of the time, you know. (Participant 4)

Just little things like that, but it fluctuates from day to day. He generally has more good days than bad, but he does, I would say, half of the week’s good and maybe 3 days of the week he’ll. . . he’s not so good. Where he is sleepy, and he just can’t be bothered with anything. (Participant 5)

And it can be sort of, you know, maybe he could be alright in the mornings and then in the afternoon, he will get confused. (Participant 2)

Additionally, caregivers also reported that there were inconsistencies in the perceived duration of the cognitive fluctuations:

It will be a few minutes, I think. It depends what it is. If it’s a long drawn out thing then it could go on for the full length of whatever. So, if you’re doing something, you’ll get her on for the full length of it, up until her concentration is took on to something else. (Participant 1)

Caregivers highlighted that the frequency of cognitive fluctuations increased in line with the duration of time since the dementia diagnosis:

Well, I mean they might seem not that often, but definitely more often than before. About how many times a day? I would say, it is hard to say actually, but I would say, something like 5, 6, 7 times a day. (Participant 1)

This had an impact upon activities of daily living for both the individual living with DLB, as well as their caregivers. As a related point, one caregiver reported that they found it very difficult to judge the severity of cognitive fluctuations, when they occurred. The caregiver found it particularly difficult to judge this when the individual with DLB did not share their impact with the caregiver:

Yeah, it’s a difficult question really, because sometimes I think she doesn’t tell me her true feelings sometimes. (Participant 6)

Overall, it is evident from these data that the experiences of cognitive fluctuations varied between participants, with no common patterns.

### Sub-theme 2: Triggers affecting symptom variation

The perceived influence of subjective sleep disturbances, such as poor subjective sleep quality, upon cognitive fluctuations was judged to be less clear by caregivers. That said, some caregivers believed that poor nocturnal subjective sleep quality was potentially a specific trigger for the subsequent worsening of cognitive fluctuations:

Oh aye, definitely, sleep, aye, aye. When she has had not so much of a good night, you know [she experiences cognitive fluctuations]. (Participant 1)

However, when sleep was discussed in this context by the caregiver, it was not consistently identified as a trigger. Overall, it appeared to be difficult for caregivers to judge whether or not subjective sleep might have had an impact upon this symptom:

No, I know sleep does, but when you look back on the episodes that they happen, there’s no real trigger, it is just a matter of trying to explain something and then we just lose what we were talking about. (Participant 1)

No, I think the only trigger is if he’s over done it the day before, I know he is not going to have such a good day the next day, where he is more lethargic and he’s less talkative, but his sleep, his sleep is really good. (Participant 5)

## Theme 2: Patient and caregiver sleep disturbances

Another theme was that caregiver participants reported that various subjective sleep disturbances were present in the individuals with DLB. The nature of the sleep disturbances which were experienced were reported to be heterogenous, and there were no consistent patterns in experiences between participants, with the exception of excessive daytime sleepiness (which is in keeping with the high clinical prevalence of this symptom^14^). As part of this theme, caregivers also described how living with an individual with DLB negatively impacted their own subjective sleep quality and sleep experience.

### Sub-theme 1: Variation in sleep disturbances

Several caregivers described how individuals with DLB experienced a wide range of subjective sleep disturbances:

You think she’s getting the same amount of sleep really. It is not getting up early, it is not going to bed later. But, more disturbed, you know. And she will say she’s tired. (Participant 1)

There was a great deal of variety in the nature and frequency of the symptoms, and in their impact upon the individual with DLB:

Well, no, sometimes she is fine, you know, you’ve just got these days where these things happen, where she has had more disturbed sleep. They are not, it is not every night. (Participant 1)

Caregivers also highlighted the occurrence of nocturnal awakenings, and of potential clinical sleep disturbances including sleep apnoea and restless legs syndrome:

Well [anonymised] has got sleep apnoea and he has had that for a good few years [. . .] And he had an appointment at the sleep apnoea clinic, and I told them how [anonymised] was struggling with the machine he had to wear to at night. (Participant 2)

He has this huge problem with restless leg syndrome. (Participant 7)

These additional clinical sleep symptoms often resulted in associated sleep disturbances, such as nocturnal awakenings:

But as I say, at that stage, he was getting up an awful lot through the night. (Participant 2)

### Sub-theme 2: Excessive sleeping

Another theme which was apparent from caregiver interviews concerned the high prevalence of daytime sleep/napping, and excessive daytime sleepiness, in people with DLB. This excessive daytime sleepiness was common across the sample and was judged to occur by caregivers, even when the individual had recently woken up:

He takes the dog out for a walk, but in between that, he could still drop off to sleep, before he gets up, you know, and I’ll say to him, come on, wake up, you’ve only just got up. (Participant 4)

Interestingly, despite excessive daytime sleepiness being a common DLB symptom, the caregivers considered this to occur as a result of old age, or poor nocturnal sleep quality, rather than because of the dementia itself. For example, Participant 4 considered this to be the case:

He will say, oh my God, he said, I had the terriblest, terriblest nightmare [. . .] I am just wondering, because they are so disturbed his dreams whether that is why he’s so tired during the day. (Participant 4)

Obviously his old age. . . you know, everyone kind of starts wanting to have naps. (Participant 4)

It was difficult for participants to identify a particular trigger for excessive sleepiness. Participant 7 described the ‘*difficulty*’ of living with the individual with DLB, as they constantly fall asleep:

In the evening, if we’re watching TV, he will start watching something and either he just can’t keep up to pace with the plot on whatever we’re watching or quite often, he just falls asleep during it or if during the day we’re just sitting around, he will fall asleep when we’re in the garden. He goes to sleep a lot, so he keeps having naps during the day. He’ll go off, sometimes for half an hour, sometimes for less, but will regularly, right over the day, fall asleep. And in the morning when he wakes up, it takes a long time to get up. (Participant 7)

### Sub-theme 3: Impact of DLB upon caregiver subjective sleep

Caregivers also reported that their own subjective sleep was negatively impacted by the effect of DLB. In particular, caregivers reported that this was because of the sleeping behaviours exhibited by people with DLB, particularly during the night:

I have never been a brilliant sleeper anyway, so I can’t say it’s all totally [anonymised’s] fault, but a big impact really on my sleep and even now, even though [anonymised] sleeps most of the night. (Participant 2)

I know he probably gets up on average, I will hear him up maybe 2–3 times through the night. (Participant 5)

Some caregivers stated that they actively listened for overnight actions and behaviours, and that this affected their own subjective sleep:

My sleep is interrupted, just when he gets up, but I’m one of these people that could sleep on a clothesline and I just go straight back to sleep. (Participant 5)

Caregivers also highlighted that sleeping in separate bedrooms was common, even when the individuals with DLB, who was typically their spouse, did not report having any sleep problems:

I don’t stay, sleep in the same room as [anonymised]. (Participant 4)

And that’s why he sleeps separately. Because he doesn’t want to disturb me. And [sighs], I’m in. . . you know, I’ve found it very hard suddenly, you know, after all these years to be sleeping by myself. (Participant 7)

It is notable that caregivers reported that their subjective sleep disturbances had a detrimental effect upon their own well-being. Participant 7 described the difficulty of being a caregiver whilst being able to obtain only a relatively short duration of nocturnal sleep:

But at the same time, it was very hard for me doing all the caring stuff during the day and basically functioning on, you know, over all 2 – 4 h’ sleep a night. (Participant 7)

## Discussion

The main aim of this qualitative study was to explore the phenomenology of cognitive fluctuations in DLB. The secondary aims of the study were to examine if caregivers considered sleep, or sleep disturbances, to influence the nature of cognitive fluctuations, and to also understand the nature of habitual sleep patterns, sleep changes and sleep disturbances, and their impact upon people with DLB and their caregivers.

The main findings of the study indicated that there was a great deal of individual variation in both the frequency and duration of cognitive fluctuations, and in their severity, in people with DLB. Patient sleep disturbances were very common and heterogenous, although not all caregivers reported that poor subjective nocturnal sleep was present. Daytime napping, and excessive daytime sleepiness, was extremely common. An additional point in relation to this was that some caregivers reported that their own sleep was negatively affected where people with DLB had sleep disturbances, partly due to actively listening for any overnight events and behaviours. For other caregivers, the patient sleep disturbances resulted in them needing to sleep in separate bedrooms. However, caregivers did not all consistently report that the patient’s sleep experience appeared to affect the degree or nature of their subsequent cognitive fluctuations.

A common theme was that caregivers reported that the nature of cognitive fluctuations greatly varied between individuals, in terms of their frequency, duration and severity. This is in keeping with the observation that cognitive fluctuations are very difficult to define.^6,28^ It is not known if there are individual variations in their periodicity and/or severity,^12^ but overall, this suggests that that the nature of cognitive fluctuations is highly likely to differ between individuals. This provides a clear starting point for future studies, which should investigate inter-individual and intra-individual differences in cognitive fluctuations, as this may improve the accuracy of subjective measurement tools. As a related point, it is notable that one caregiver reported that they found it difficult to judge the severity of cognitive fluctuations when they occurred and when the individual with DLB did not share their impact. As commonly used clinical questionnaire measures rely on caregiver responses,^16^ future work might wish to examine if this could potentially impact their reliability.

Caregivers reported that qualitatively, a range of sleep disturbances were apparent in people with DLB, which included nocturnal awakenings, sleep apnoea, restless legs syndrome, nightmares and symptoms of REM behaviour disorder. This is in keeping with the observation that clinically, a wide range of sleep disturbances are highly prevalent in DLB.^29,30^ Excessive daytime sleepiness was also highlighted as a troublesome symptom which had an impact upon patients; this is also a symptom which, based on some studies, can occur in up to 100% of people with DLB.^14^

An important finding from this study, with clinical relevance, is that caregivers reported that patient symptoms had an impact on their own subjective sleep experience, primarily caused by actively listening, or at least being aware, of overnight events. This is likely to negatively affect caregiver sleep: even healthy sleepers can display an increase in sensory processing, and sleep disturbances, in a noisy environment.^31^ Additionally, this may cause an increase in pre-sleep cognitive activity, which refers to being worried or attaining or maintaining sleep, being unable to prevent thoughts occurring, or having a racing mind prior to sleep, and this can also negatively affect subjective sleep.^31^ As insufficient sleep duration and quality are associated with a range of negative physical and psychological health outcomes,^32,33^ these qualitative results suggest that DLB caregivers may be at particular risk of sleep disturbances. This is important as caregivers who experience sleep disturbances may themselves have an increased risk of cognitive impairment or dementia.^34,35^ Future studies should focus on understanding the sleep of DLB caregivers, and potentially design bespoke sleep interventions which are designed to maintain or improve sleep health.

Qualitatively, the impact of sleep upon cognitive fluctuations appeared to be inconsistent. This may be because of the wide individual heterogeneity which was apparent in this symptom. Alternatively, although there are potential underlying mechanisms common to sleep and cognitive fluctuations,^7,14^ this may be difficult to judge qualitatively. Specifically, caregivers were being asked to provide a subjective, qualitative assessment of the patient’s sleep experience: sleep is a complex physiological process which can be assessed using subjective and/or objective measurement methods^36^ and sensitive quantitative sleep measurement methods may be needed to confirm whether or not sleep has an impact upon this symptom.

The main strength of the study is that we recruited primary caregivers for individuals with DLB who were participating in an ongoing research study, and participants had a diagnosis of probable DLB, and not a self-reported diagnosis. This is important because DLB is frequently misdiagnosed.^37^ One limitation of this study is that we only recruited a relatively small sample of DLB caregivers from the north-east of England, as it was not possible to determine the sample size in advance due to the pilot nature of this study, since we recruited caregivers of DLB participants who were taking part in a larger research study. However, during recruitment for the study, we did not specifically target caregivers who reported that the person for whom they cared for had severe cognitive fluctuations or severe sleep disturbances. As we have no reason to believe that our DLB caregivers are not representative of a wider sample of caregivers, it is very unlikely that this has affected the overall results. However, we strongly encourage that future studies use a larger sample of caregivers, wherever possible.

Another limitation of this study is that although the interview questions were directly informed by feedback provided by DLB caregivers, the questions were not fully pilot tested in advance of this study. Consequently, we recommend that where the resources are available, that future studies pilot test interview schedules to ensure their suitability in order to aid participant understanding; this is likely to be useful in the context of symptoms which can be difficult to define. Although it may be a limitation that interviews were conducted by telephone, as in-person interviews may have aided the understanding of caregiver participants, it should be noted that interviews took place during the COVID-19 pandemic. At the time of data collection, both caregivers and people with DLB were considered to be vulnerable and at a high risk of infection. Therefore, the use of telephone interviews aided recruitment from a feasibility perspective. Indeed, one intervention study which was conducted during the COVID-19 period found that a remote intervention was useful for treating individuals who had a fear of infection, and as an additional advantage, social distancing measures (intended to prevent the spread of COVID infection) were not required.^38^ In this context, the advantages of conducting the study remotely far outweighed the disadvantages. As we have still demonstrated that this approach is feasible, future studies involving DLB caregivers may wish to consider whether telephone, or other remote methods of assessment, could suitably replace or supplement in-person interviews.

Another potential weakness of the study is in that caregivers were asked to self-report the nature and severity of cognitive fluctuations experienced by the individual with DLB. This may have been affected by caregiver recall bias, or by other factors including concurrent illness or fatigue.^10,12,13^ However, this potential weakness is unlikely to have affected the results of the study. This is demonstrated by previous work which has shown that that even qualitatively, fluctuations in cognition are distinctly different between DLB and AD.^6^ As a related point, because subjective questionnaire measures of cognitive fluctuations, which are very frequently used in both research and clinical environments, also rely upon retrospective caregiver estimates of their frequency and severity,^16^ this limitation is not unique to this study.

## Conclusion

Overall, the main findings from this study are that qualitatively, caregivers report that DLB cognitive fluctuations show large variations in terms of their frequency, duration and severity. A range of sleep disturbances are also commonly observed by caregivers, including excessive daytime sleepiness, nocturnal awakenings, restless legs and sleep apnoea. The impact of sleep upon cognitive fluctuations was not clear and suggests that the subjective sleep experience may not directly affect this symptom. Caregivers also reported that their own sleep was negatively affected by the sleep disturbances experienced by individuals with DLB, potentially as a result of caregivers actively listening for overnight events. Together, these results indicate that better measurement tools are needed for cognitive fluctuations, and interventions are required to maintain good sleep health in DLB caregivers.

## Supplemental Material

sj-docx-1-smo-10.1177_20503121241271827 – Supplemental material for Understanding the nature and impact of cognitive fluctuations and sleep disturbances in dementia with Lewy bodies: A qualitative caregiver study Supplemental material, sj-docx-1-smo-10.1177_20503121241271827 for Understanding the nature and impact of cognitive fluctuations and sleep disturbances in dementia with Lewy bodies: A qualitative caregiver study by Ellie Matterson, Gemma Wilson-Menzfeld, Kirsty Olsen, John-Paul Taylor and Greg J Elder in SAGE Open Medicine

sj-docx-2-smo-10.1177_20503121241271827 – Supplemental material for Understanding the nature and impact of cognitive fluctuations and sleep disturbances in dementia with Lewy bodies: A qualitative caregiver study Supplemental material, sj-docx-2-smo-10.1177_20503121241271827 for Understanding the nature and impact of cognitive fluctuations and sleep disturbances in dementia with Lewy bodies: A qualitative caregiver study by Ellie Matterson, Gemma Wilson-Menzfeld, Kirsty Olsen, John-Paul Taylor and Greg J Elder in SAGE Open Medicine
