# Supplementary material for: Understanding the nature and impact of cognitive fluctuations and sleep disturbances in dementia with Lewy bodies: A qualitative caregiver study
Source: SAGE Open Med. 2024 Oct 9;12:20503121241271827. doi: 10.1177/20503121241271827 (PMC11468633; doi:10.1177/20503121241271827)
Supplement: sj-docx-2-smo-10.1177_20503121241271827 – Supplemental material for Understanding the nature and impact of cognitive fluctuations and sleep disturbances in dementia with Lewy bodies: A qualitative caregiver study [file sj-docx-2-smo-10.1177_20503121241271827.docx]

*Supplementary Material 1: Interview schedule*

1. Please tell me what you think their [the participant’s relative/family member with DLB] cognitive fluctuations are like? Do they feature ‘change in awareness’, ‘reduction in alertness’, ‘short episodes of confusion’ and ‘communication difficulties’? Could you describe a recent, or past, experience of this?
2. In your experience, please can you tell me about how often the cognitive fluctuations happen, and how long they go on for? When do they happen? Is there anything that comes before these episodes- any specific events, bad sleep, triggers that seem to impact them? Could you describe a recent, or past, experience of this?
3. Please could you tell me how you think cognitive fluctuations affect them? Could you describe a recent, or past, experience of this?
4. Please could you tell me how you think cognitive fluctuations affect you? Could you describe a recent, or past, experience of this?
5. Please could you tell me what you think about their sleep? Do they have any sleep problems? Do these problems impact their day-to-day life? Could you describe a recent, or past, experience of this?
6. Please could you tell me if their sleep, or sleep difficulties impact your sleep? Could you describe a recent, or past, experience of this?
